# Feasibility, Barriers, and Facilitators of Long-Term Physical Activity Tracking During Treatment: Interview Study Among Childhood Cancer Patients

**DOI:** 10.2196/75322

**Published:** 2025-08-27

**Authors:** Emma den Hartog, Wim J E Tissing, Sebastian B B Bon, Patrick van der Torre, Emma J Verwaaijen

**Affiliations:** 1 Department of Suportive Care, Princess Máxima Center for Pediatric Oncology, Heidelberglaan 25, Utrecht, 3584CS, The Netherlands, 31 0889727272; 2 Department of Pediatric Oncology and Hematology, Beatrix Children’s Hospital, University Medical Center Groningen, University of Groningen, Groningen, The Netherlands

**Keywords:** physical activity, tracking, smartwatch, pediatric oncology, feasibility

## Abstract

**Background:**

Children with cancer are at risk of reduced physical activity. Gaining insight into physical activity using smartwatches could improve understanding of individual potential during treatment, support early recognition of aberrant physical activity, and enable tailored support.

**Objective:**

This study aimed to explore the feasibility, barriers, facilitators, and considerations of long-term physical activity tracking using a smartwatch during childhood cancer treatment.

**Methods:**

In this prospective study, 30 children (age 8‐18 years) under active cancer treatment were included in 2 phases. During phase 1, 15 children wore a smartwatch daily for 12 consecutive weeks, and in-depth interviews were conducted to identify principal considerations used to optimize wearability and the methods for phase 2. In phase 2, another 15 children wore the smartwatch, and semistructured interviews were conducted at weeks 1, 3, 6, and 12. These interviews were thematically analyzed to identify barriers and facilitators. An iterative process of alternating data collection and analysis allowed for ongoing method refinement and deepening thematic analysis during the study period.

**Results:**

Key considerations for improvement identified in phase 1 led to refinements in phase 2, including enhanced engagement, regular prompts, customized plans, personalized setup, and improved aesthetics and comfort. The interviews conducted during phase 2 identified barriers and facilitators. The 4 most prominent themes were burden and resilience, motivational drivers and perception, insight and evaluation, and user experience and functionality. Feasibility was influenced by the child’s physical state and perceived burden. Motivation, perceived value, and expectations played crucial roles in sustaining adherence, while also the balance between positive reinforcement and potential confrontation affected long-term use. User experience, including attractiveness, comfort, and usability, impacted acceptance.

**Conclusions:**

Real-time and long-term physical activity tracking using a smartwatch in children during cancer treatment was not feasible in our cohort. A personalized approach, incorporating individual preferences and physical condition, is essential to support adherence.

## Introduction

The overall 5-year survival of childhood cancer has improved tremendously over the past decades, reaching around 80% [1-3]. Despite this improvement, pediatric cancer and its treatment are often accompanied by adverse health effects, such as muscle deficits, pain, and fatigue, that negatively influence physical functioning [1,4,5]. Physical activity may help mitigate these adverse health effects by improving body composition, muscle strength, flexibility, and cardiorespiratory fitness and subsequently enhancing physical functioning and quality of life [6-8].

However, children with cancer may experience physical weakness, limited opportunities to engage in physical activity, and a shift in focus due to emotional strain because of their disease, its treatment, and other medical needs [9-12]. Although it is well established that children with cancer are at risk of reduced physical activity [5,10,11,13], there is limited knowledge about variation in physical activity levels both between and within children during treatment. These variations depend on the child’s diagnosis, treatment type and intensity, and medical complications, as well as personal and environmental factors [11,12,14,15]. Gaining insight into physical activity levels could improve understanding of children’s physical potential during different treatment phases, support early recognition of aberrant physical activity levels, and enable tailored advice and guidance to promote physical activity that aligns with the child’s needs, their family, and the treatment phase they are in. Understanding variations in physical activity levels requires accurate and continuous measurement of physical activity.

Currently, the most commonly used methods to monitor physical activity levels in children with cancer include questionnaires and accelerometry devices [10-13,16]. Questionnaires are prone to subjective inaccuracies, as children and parents have to recall and may under- or overestimate their physical activity [17]. Accelerometry addresses these limitations but has notable drawbacks that restrain usability, including the need for installation to collect data, the lack of real-time accessible data for children, parents, and health care professionals, and a nonmodern design that does not offer visual feedback to the user. Furthermore, objective devices have primarily been used for short-term monitoring (1‐2 weeks) [10,11,13,15,18], which limits their ability to capture variations in physical activity levels across different treatment phases of childhood cancer.

Consumer-level smartwatches are increasingly being used in health care and research to track physical activity [19-25]. A major advantage of smartwatches is that they provide insight into real-time data and visual feedback for the patient. Their use during treatment for childhood cancer would enlarge knowledge about physical activity levels during different treatment phases and therefore support both patients and their health care professionals in managing expectations of physical activity. Moreover, using smartwatches to track physical activity would facilitate early recognition of children with aberrant physical activity levels and enable timely initiation of interventions to prevent aggravation. In children with a brain tumor who received an intervention, including the use of a smartwatch combined with coaching by a physiotherapist for 12 weeks during treatment, no adverse effects of using the smartwatch were reported [26]. In addition, children with acute lymphoblastic leukemia were able to use a fitness tracker clipped on clothing for around 3 weeks during their treatment, consistently with low effort [27]. During the maintenance phase of childhood acute lymphoblastic leukemia and after treatment for childhood cancer, the daily use of a smartwatch for 6 months was feasible as measured by retention, receptivity, and belief of utility [28]. However, in healthy children and adolescents, several issues concerning the use of smartwatches, including technical difficulties, a novelty effect, and lack of comfort, were identified, which limit long-term use [29,30]. There is limited knowledge about the feasibility and wearing experiences of long-term physical activity tracking using smartwatches during different phases of childhood cancer treatment [31].

Therefore, the aim of this study was to determine the feasibility and to identify barriers, facilitators, and key considerations of real-time and long-term physical activity tracking using a consumer-level smartwatch in children during cancer treatment. We hypothesized that physical activity tracking using a smartwatch in children undergoing cancer treatment would be feasible and imagined that factors, including treatment phase, medical needs, and personal desires, could hamper long-term use.

## Methods

### Study Design

This prospective study consisted of 2 phases in which quantitative data and qualitative data were collected to determine feasibility and to identify barriers, facilitators, and key considerations for long-term physical activity tracking in children with cancer during treatment, respectively.

### Participants and Inclusion

Children aged 8‐18 years, diagnosed with a malignancy, and undergoing active treatment in the Princess Máxima Center for Pediatric Oncology were eligible for participation. Inclusion criteria were the ability to walk at least 50 meters without aid, adequate understanding of the Dutch language, and access to a digital device capable of downloading an app (eg, a smartphone). Exclusion criteria included not being able to attend evaluation moments, undergoing stem cell transplantation during the study period, and pre-existing intellectual disabilities that prevented participation in interviews or long-term smartwatch use.

Potential participants were recruited by the researcher (EdH) in collaboration with their pediatric physiotherapist or oncologist.

### Ethical Considerations

Written informed consent was obtained from participants aged ≥12 years and parents or caregivers of participants aged 8‐16 years. The Medical Research Ethics Committee (MREC) confirmed this study was not subject to the Medical Research Involving Human Subjects Act (23-003). Data were pseudonymized. Participants were not compensated.

### Study Procedure

In phase 1, conducted between May and September 2023, 15 children undergoing active cancer treatment were asked to wear a smartwatch daily for 12 consecutive weeks. A time frame of 12 weeks was chosen, as this was the first effort to explore the potential of a smartwatch to track long-term physical activity in children with cancer during different phases of treatment. Moreover, we expected that if wearing the smartwatch was feasible for 12 weeks, it would remain so for a longer period. In addition, within 12 weeks, they usually undergo several treatment courses that vary in type and intensity, allowing them to experience the smartwatch during different phases of treatment. After 12 weeks, in-depth interviews were conducted to identify key considerations for long-term physical activity tracking during childhood cancer treatment. These findings were then used to optimize the methods for phase 2.

In phase 2, conducted between February and June 2024, another 15 children wore the smartwatch for 12 consecutive weeks. After having optimized the methods for phase 2 during phase 1, total wear time was calculated, and semistructured interviews were conducted at weeks 1, 3, 6, and 12 during phase 2. The total wear time and interviews were used to evaluate feasibility and identify barriers, facilitators, and key considerations. The iterative approach of alternating data collection and analysis enabled ongoing method refinement based on children’s experiences, while incorporating emerging topics and deepening thematic analysis during the study period.

Upon inclusion, children were instructed to wear the smartwatch daily during the upcoming 12 weeks. An app (Health Mate, Withings) was installed on a smartwatch or tablet of children or their parents. The smartwatch was linked to an account created in the app, which enabled the researcher to gain insight into physical activity data, which was transferred to a dashboard developed by the Information Technology, Data Provision, and Technology department of the Princess Máxima Center. In the app, physical activity, including steps and heart rate, was displayed for children themselves. Children received a notification on the smartwatch and in the app when they reached their self-chosen, adjustable daily step goal. Moreover, during phase 2, children or their parents received prompts with feedback on wearing behavior and tips for smartwatch use. These prompts were sent as text messages to their phones or via emails every other week.

### Smartwatch

In this study, children wore a Withings (Withings, Issy-les-Moulineaux, France) Steel Heart rate (HR) (Model: HWA03b-36black/white-Inter) or Pulse HR (Model: WAM03-Black Mirror-All-Inter) smartwatch. The model for each child was determined by the researcher in agreement with the child to optimize wearability. Generally, larger children were assigned a Steel HR, while those with a slim wrist received a Pulse HR. The device measured step count and HR and was able to track a variety of sport activities. Withings smartwatches have been used in a number of previous clinical studies as a method to evaluate physical activity [20,21,23,25]. Also, in healthy children (8‐11 years old), a Withings smartwatch has previously been used to evaluate an intervention aiming to increase physical activity [22]. In healthy adults, Withings smartwatches have been validated against a reference device (ActiGraph) for counting steps [32]. Among a set of smartwatches, these were found to be the most user-friendly, satisfactory, accurate, and repeatable for tracking steps [33] and showed excellent test-retest reliability (intraclass correlation: 0.92, 95% CI 0.83‐0.96, p<0.01) [34].

### Data Collection

#### Feasibility

Feasibility was evaluated by acceptance (ie, the percentage of eligible participants who agree to take part after being invited), attrition (ie, the percentage of participants who remain in the study until the end), and adherence (ie, the total wear time) [35]. Adherence was examined only in the 15 children included in phase 2 after 12 weeks of wearing the smartwatch, as the goal of phase 1 was to optimize the methods for phase 2. We defined that the adherence feasibility target for long-term physical activity tracking in children with cancer during treatment was reached when at least 60% (9/15) of the children in phase 2 adhered to wearing the smartwatch for a minimum of 75% of the study period. This target was based on previous studies using activity trackers in childhood cancer survivors [28,36,37].

#### Key Considerations, Barriers, and Facilitators

Key considerations for improvement, along with barriers, facilitators, and future key considerations of long-term physical activity tracking during childhood cancer treatment, were identified through in-depth interviews at the end of phase 1 and semistructured interviews during phase 2, respectively.

During the interviews, an interview guide was used that covered topics, including reasons for wearing or not wearing the smartwatch, barriers and facilitators, and children’s thoughts on wearing it for the entire treatment and sharing their physical activity data with their physiotherapist. The interview guide for the in-depth interviews was developed based on previous studies and experiences raised during phase 1, while the interview guide for the semistructured interviews was constructed and adjusted according to findings of phase 1 and experiences during phase 2. The interview guides used in both study phases are shown in Sections A and B in Multimedia Appendix 1, respectively. All interviews were conducted by EdH and planned on the same day as participants’ medical appointments at the Princess Máxima Center, or they were held via a phone or video call to avoid extra study visits to the hospital. For children aged <12 years, children themselves and their parents were interviewed. Children aged ≥12 years were interviewed themselves or with the help of their parents, depending on the family’s preferences. In case of questions or issues between the evaluation moments, participants contacted the researcher via email or phone.

### Data Analysis

Descriptive statistics were used to describe patient characteristics, acceptance, and attrition. Categorical variables were presented as numbers with percentages. Continuous variables were presented as mean with SD for normally distributed data or as median with IQR for non-normally distributed data. Analyses were performed in IBM SPSS Statistics version 30.0.0.

To determine adherence, the total wear time was calculated as a percentage by dividing the total number of valid days children wore the smartwatch by the total number of days a child was included in the study. A day was considered valid when HR was measured at least once per hour for a minimum of 8 hours between 6 AM and 12 PM.

The interviews were recorded on a mobile phone and transcribed verbatim. The in-depth interviews from phase 1 were analyzed using Braun and Clarke’s [38] thematic approach. Transcripts were initially coded by EdH, and the final codes and themes were formulated in discussion with EJV and SBBB. Themes were subsequently discussed within the entire research team to identify key considerations that were used to guide the development of phase 2.

The interviews from phase 2 were analyzed using Braun and Clarke’s [38] inductive thematic approach, which included an iterative process of data collection and analysis [39,40]. The first step involved reading interview transcripts to familiarize yourself with the data. In the second step, EJV and EdH independently coded 12 transcripts. These codes were discussed with SBBB, resulting in a preliminary thematic map, which EdH used in the remaining transcripts. In the third step, subthemes and themes were collaboratively established by SBBB, EJV, and EdH and further discussed within the entire research team. The last step involved finalizing the codes, subthemes, and main themes while identifying connections within and across themes. The thematic analysis was conducted using ATLAS.ti (Scientific Software Development GmbH, v8.3.0).

## Results

### Cohort

Most of the 15 children included in phase 1 were male (11/15, 73%) and diagnosed with a hematological malignancy (13/15, 86%) (Table 1). They had a mean age of 12.2 (SD 2.9) years at the time of inclusion, and the median time since diagnosis was 262.0 (IQR 108.5‐325.5) days. The acceptance and attrition rates were 45% (15/33) and 93% (14/15), respectively. Reasons for refusing participation included already owning a smartwatch, unwillingness to wear a smartwatch, disliking the smartwatch’s feeling or appearance, not perceiving the need for a smartwatch to measure physical activity, and considering the smartwatch as an additional burden alongside medical needs.

Of the 15 children included in phase 2, 7 (47%) were male, and 12 (80%) were diagnosed with a hematological malignancy (Table 1). Children had a mean age of 12.5 (SD 2.9) years at time of inclusion, and the median time since diagnosis was 96.0 (IQR 80.5‐151.0) days. The acceptance and attrition rates for phase 2 were 83% (15/18) and 93% (14/15), respectively. Reasons for refusing participation included unwillingness to wear a smartwatch that measures physical activity during treatment and parental concerns related to their child’s autism.

An overview of the number of dropouts and patients included in quantitative and qualitative data analyses is shown in Table 1. During both study phases, 1 child dropped out because wearing the smartwatch was considered too much of a burden in combination with medical needs. All available data of these children were used for data analysis, and no new children were recruited. In 71% (10/14) of the in-depth interviews at the end of phase 1, parents were present during the interview. During phase 2, parents were involved in 79% (11/14), 73% (11/14), 50% (6/12), and 71% (10/14) of the semistructured interviews at weeks 1, 3, 6, and 12, respectively.

**Table 1.** Patient characteristics at time of inclusion and an overview of the number of dropouts and children included in quantitative and qualitative data analyses during study phase 1 and 2.

Study phase	Phase 1 (n=15)	Phase 2 (n=15)
Patient characteristics at time of inclusion		
Sex, n (%)		
Female	4 (27)	8 (53)
Male	11 (73)	7 (47)
Age (year), mean (SD)	12.2 (2.9)	12.5 (2.9)
Time since diagnosis (days), median (IQR)	262.0 (108.5-325.5)	96.0 (80.5-151.0)
Diagnosis, n (%)		
Acute lymphoblastic leukemia	8 (53)	7 (47)
Lymphoblastic lymphoma	5 (33)	3 (20)
Hodgkin lymphoma	0	1 (7)
Langerhans cell histiocytosis	0	1 (7)
Medulloblastoma	0	3 (20)
Osteosarcoma	1 (7)	0
Ewing sarcoma	1 (7)	0
Weight-to-height *z*-score, mean (SD)	0.3 (1.2)	0.1 (1.5)
Cohort overview		
Acceptance, n included/n eligible approached (%)	15/33 (45)	15/18 (83)
Dropouts, n (%)	1 (7)	1 (7)
Children included in quantitative data analysis, n (%)	—^a^	13 (87)
Adherence, median proportion (IQR)	—	68 (39-92)
Children included in qualitative data analysis, n (%)	14 (93)	15 (100)
In-depth interview	14 (100)	—
Semistructured interview after 1 week	—	15 (100)
Semistructured interview after 3 week	—	14 (93)
Semistructured interview after 6 week	—	12 (80)
Semistructured interview after 12 week	—	14 (93)
Model smartwatch, n (%)		
Steel HR^b^	10 (67)	10 (67)
Pulse HR	5 (33)	5 (33)

a Not applicable.

b HR: heart rate.

### Phase 1

The in-depth interviews highlighted that children were generally willing to wear the smartwatch during cancer treatment, as it motivated them to remain physically active and achieve their daily activity goal. Parents mentioned that the smartwatch provided valuable insights into their child’s symptoms linked to physical activity. However, some children found the smartwatch occasionally annoying or burdensome. The primary reasons for not wearing the smartwatch were having a lack of knowledge about potential benefits or a sense of relevance for its use, having a lack of stimulating environment, and not perceiving it as beneficial, especially when not engaging in physical activity. Moreover, children did not feel attached to the smartwatch, forgot to wear it due to not being used to wearing a watch, had a lack of external assistance, and had no desire to wear it or had issues with their preferences or comfort. Quotes from the in-depth interviews emphasizing the barriers for not wearing the smartwatch mentioned by children and their parents are reported below.

*It would help wearing the smartwatch more often when showing us the usefulness. For example, we only really realized how useful the smartwatch was once he started wearing it.*

[Father of an 11-year-old boy]

*Sometimes during the day when I did not do anything, I either forgot the smartwatch or I thought “Well, yes, I am not really doing anything right now thus it is not so much use*.”

[17-year-old boy]

If you have worn the smartwatch continuously, you get…

*Father: …maybe something like a small reward. Something that shows appreciation — it does not have to be expensive or big, but something that says ‘thank you for your help’. A card, a little thing, just something — yeah, I think that would be motivating.*

[9-year-old boy and his father]

*I only took the smartwatch off because it irritated my skin. Maybe a fabric strap would be more comfortable.*

[13-year-old girl]

Based on the barriers to wearing identified in phase 1, several key considerations were identified and implemented to optimize phase 2 (Table 2). The considerations were structured into the following points:

(1) Enhanced engagement: When children received the smartwatch, they were extensively informed about the importance of wearing it, personal benefits, and its features. The information provided by the researcher was tailored to individual children. If a child, for example, was eager to move a lot, the benefits of seeing its own physical activity were emphasized. Parents were actively engaged in the process to ensure support and understanding.

(2) Regular prompts: Children or their parents, depending on the family’s preferences, received prompts with feedback on wearing behavior and tips for smartwatch use as a text message on their phone or via email. Such prompts looked like:

*When you start moving, your muscles and bones become stronger, making it easier to stay physically active. Tip: You can track your heart rate over the day, week, or month in the application by clicking on ‘Heart Rate.’ Did you know your heart rate goes up when being physically active?*

These prompts were sent after the first week of wearing and subsequently every other week. Prior research has demonstrated that such prompts were effective in maintaining engagement in children with acute lymphoblastic leukemia [41] and sustaining health-enhancing behaviors in adolescents [42,43].

(3) Customized plans: To address practical challenges, a personalized plan was created with input from children to ensure the smartwatch was not forgotten. Examples of issues addressed in such a personalized plan included placing the smartwatch on the child’s bedside table or next to the child’s smartphone during the night and putting it on directly after waking up. Parents were encouraged to assist their child in wearing the smartwatch daily if needed by giving them an active role in incorporating the smartwatch into the daily routine.

(4) Personalized setup: The smartwatch was tailored to each child’s preferences. If desired, features, such as phone call and message notifications were activated, and a daily step goal was set.

(5) Improved aesthetics and comfort: To meet individual preferences and address potential irritation, children were provided with a variety of wristbands in different colors and made of different materials.

**Table 2.** Principal considerations for long-term physical activity tracking during childhood cancer treatment gathered during phase 1 and used to optimize phase 2.

Key considerations and barriers for wearing	Specific considerations
Enhanced engagement	
Lack of knowledge about potential benefits and relevance	Provide extensive information on the benefits and relevance of wearing the smartwatch, also when not being physically active
No perceived benefit	Provide information regarding potential benefits of wearing and using the smartwatch, with a focus on benefits for children themselves
Lack of stimulating environment	Increase social support and engage parents
Regular prompts	
No attachment	Send 2 weekly prompts, including feedback on wearing behavior and tips for smartwatch use
Customized plans	
Forgotten to wear	Create a plan to not forget the smartwatch using input from children themselves to optimize wearability
Lack of external assistance	Encourage parents to assist children in wearing the smartwatch
Personalized setup	
No desire to wear	Activate incoming phone calls and messages and set a daily step goal
Improved aesthetics and comfort	
Issues with preferences or comfort	Offer a variety of wristbands in different colors, which are made of different materials

### Phase 2

Technical difficulties while synchronizing the smartwatch with the app occurred in 2 children, resulting in incomplete data retrieval. Therefore, these children were not included in the calculations of adherence. One child dropped out after 36 days because wearing the smartwatch was considered too much of a burden in combination with medical needs but was still included in the analysis. Thirteen children had a median adherence of 68 % (IQR 39%‐92%) of total days included in the study (Table 1). Overall, 6/13 children (46%) adhered to wearing the smartwatch for a minimum of 75% of the 12-week study period and reached the adherence target.

All interviews performed during phase 2 were analyzed according to an iterative approach to define themes during as well as after the study period. These interviews identified key barriers and facilitators of real-time and long-term physical activity tracking using a smartwatch in children during cancer treatment. The most prominent themes were (1) burden and resilience, (2) motivational drivers and perception, (3) insight and evaluation, and (4) user experience and functionality (Table 3). These themes are elaborated in the following paragraphs and supported with quotes from children and parents.

**Table 3.** Barriers and facilitators of real-time and long-term physical activity tracking using a smartwatch in children during cancer treatment.

Main themes, subthemes, and codes	Barrier	Facilitator
Burden and resilience		
Physical state		
Being fatigued	Feeling too exhausted during admission or intensive treatment courses.	—^a^
Feeling ill	Children find the smartwatch too burdensome when feeling sick, nauseous, and unwell.	—
Low resilience	It is too much and burdensome to wear the smartwatch on top of medical needs, such as taking medication, being hospitalized, having a drip line, receiving radiation, or tube feeding.	—
Perception of burden		
Overload	Children expect that the smartwatch is not something they could focus on shortly after diagnosis or have no interest in the smartwatch during intensive treatment courses and do therefore not expect to be willing to wear it during the entire treatment. For some parents, the smartwatch would feel like an overload during early treatment phases, as it would have been too much to deal with.	—
(No) pressure to wear	Some children feel pressured to wear the smartwatch continuously, which can become overwhelming.	Other children feel no such pressure to wear the smartwatch, enabling flexible use.
Not burdensome to wear		Wearing the smartwatch is considered not burdensome.
Enforcing the obligation may lead to resistance	The feeling of wearing the smartwatch being an obligation is unpleasant and would make wearing it during the entire treatment unbearable.	—
Motivational drivers and perception		
Motivation and stimulus		
(No) stimulus for physical activity	The smartwatch is no additional stimulus for physical activity for some children. Parents question the smartwatch’s ability to motivate or provide value if the child does not experience it as a stimulus.	For other children, the smartwatch is an additional stimulus for physical activity.
Stimulus despite pain	—	Despite experiencing treatment-related nerve pain, the smartwatch is a stimulus for physical activity.
Reward for sufficient physical activity	—	Willingness to wear the smartwatch for the visual reward after reaching the step goal.
Value and meaningfulness		
Attachment to the smartwatch	—	Feeling proud to wear the smartwatch.
Lack of obligation	The absence of expectation or mandate allows children to deprioritize the smartwatch. Therefore, children consider it as one of the first things they can let go.	—
Decreasing novelty	The initial enthusiasm for the smartwatch diminishes over time.	—
Not useful during inactivity	Depending on the intensity of treatment and its effect on how physically active children can be, wearing the smartwatch may not be doable for some children. Especially when children are physically inactive, such as when they feel unwell or tired, are resting, or shortly after diagnosis, they are unsure about the meaningfulness of the smartwatch.	For other children, it might be doable to wear the smartwatch during treatment with varying intensity and effect on physical activity.
Lack of intrinsic motivation	—	Some children wear the smartwatch out of habit or external expectation rather than personal interest.
Concerns around smartwatches		
Inaccurate tracking of physical activity	Children are disappointed when steps or other activities are not tracked accurately, as they want all their physical activity to be captured.	—
Parental concerns about overemphasis on physical activity	Some parents feel their child places too much focus on the smartwatch, potentially leading to excessive physical activity or obsession with activity tracking.	—
Insight and evaluation		
Self-monitoring and positive evaluation		
Tracking physical activity for self-monitoring and evaluation	—	Having insight into steps, heart rate, sleep, distance covered, calories burned, and time is convenient and used as an evaluation of the number of steps taken on different days and during and after physiotherapy consultations.
Confirmation of sufficient physical activity	—	Insight provides confirmation of being sufficiently physically active.
Insight and health evaluation by parents	—	Parents appreciate having insight, linking their child’s physical activity to its feelings or complaints, and using the smartwatch as an additional medical tool. Also for parents, the smartwatch motivates them to be physically active or helps with seeking opportunities.
Sharing insight with the environment		
Providing insight into physical activity for oneself and others	—	Children feel positive about having insight into their own physical activity and being able to share this with others. It helps them show others that being ill does not prevent them from staying physically active.
Support from a pediatric physiotherapist	—	Children find it beneficial when their pediatric physiotherapist has access to their physical activity data and supports them through encouraging messages to stay active.
Challenges with tracking and insight		
Negative feelings about tracking and external feedback	Failing to meet daily step goals can feel confronting for some children. Parents worry that such confrontation could have a discouraging effect. Furthermore, children might find it unpleasant if their pediatric physiotherapist had insight into their low physical activity levels and pointed out their physical inactivity.	Other children do not perceive it as problematic when failing to meet daily step goals.
User experience and functionality		
Comfort and fit		
Appearance	Some children find the smartwatch unattractive.	Other children consider the smartwatch attractive, appreciating features like a preferred strap color or matching it with their clothes.
Wearing comfort	Particularly shortly after diagnosis or during early treatment, wearing the smartwatch is sometimes considered unpleasant. The smartwatch sometimes leaves an imprint, presses on the skin or bone, or causes skin irritation, which makes children not willing to wear the smartwatch for the entire treatment.	For most children, the smartwatch feels comfortable and unobtrusive, and they think it is doable to wear the smartwatch throughout the entire treatment.
Skin irritation	The smartwatch can cause skin irritation, such as itching, bumps, or small lesions, particularly shortly after diagnosis, when combined with certain medications, such as treatment with glucocorticoids, or sweating.	—
Fit	The smartwatch can sometimes be too tight or too loose, shifting on the wrist.	Even the smallest smartwatch may feel oversized for young children due to their small wrists, but they may still prefer to keep it on.
Strap material	Some children think that a fabric strap is not better than a rubber strap.	Other children think that a strap made of a different material than rubber, such as metal or fabric, might be more comfortable or attractive.
Needs and preferences		
Ease of use	—	The smartwatch is considered easy to use.
Features and functionalities	—	The long battery life and waterproof design are appreciated. Preferences vary: one child prefers a smartwatch without a digital screen while another favors a fully digital display.
Preference for phone-based physical activity tracking	One child prefers to wear an attractive, nontracking watch and use a phone to track physical activity rather than wearing a less attractive smartwatch that tracks physical activity.	—
Technical functionality		
Phone notifications	Inconsistent visibility of notifications on the smartwatch can be frustrating.	Receiving phone notifications on the smartwatch is considered convenient and enjoyable.
Difficulty synchronizing with the app	When the smartwatch is not properly connected to a phone or tablet, some activity data may not be recorded in the app, limiting its usefulness.	—
Incorrect setup limits functionality	If the smartwatch screen is not set up correctly, physical activity data may not be displayed.	—
Reliability and sustainability		
Strap quality	The strap and its loops are of low quality, which is seen as inconvenient.	—
Inconsistent activity measurement	Steps or heart rate do not always seem to be measured continuously or consistently.	—

a Not available.

### Burden and Resilience

The experience of wearing the smartwatch varied widely among children, with some who found it overly burdensome, particularly during periods of fatigue, nausea, or general illness. For children with low resilience or high medical needs, the additional effort of wearing the smartwatch could be perceived as overwhelming.

*But everything that came with it, was too much for her. She no longer wanted to take medication. In the meantime, she has been given a feeding tube. She was very ill, so she had absolutely no interest in the smartwatch anymore, you know.*

[Mother of an 11-year-old girl]

The timing of introducing the smartwatch played an important role. Shortly after diagnosis or during intensive treatment phases, children may not be emotionally ready to engage with the smartwatch. Parents noted that during the initial month of treatment, the smartwatch could feel like an overload on top of an already overwhelming situation. While some children found the associated expectations and pressure of wearing the smartwatch overwhelming, making it unsustainable, others experienced no such difficulty and wore it with ease. When children had the feeling that wearing the smartwatch was mandatory and that they were not supposed to take it off, wearing it became unpleasant and unsustainable for the entire treatment.

*I could have worn the smartwatch at the beginning of the treatment, closer to the diagnosis, but I do not know if it would have been very useful, because at that time I could not really do anything. So I am not sure if it would have helped much. I think probably yes, but I do not know for sure, because I was not thinking about that back then. It was more like, “Oh shit, I cannot walk,” you know?*

[16-year-old girl]

*The thought that you are not supposed to take it off is exactly what makes you want to take it off.*

[12-year-old boy]

### Motivational Drivers and Perception

The smartwatch served as a motivator for physical activity for some children, even in the presence of pain. For others, however, it did not provide an additional motivation. Parents questioned its value when their child did not perceive it as a stimulus for physical activity.

*I wore the smartwatch to track how much I moved during the day and things like that. At the end of the day, I would check what I did — like how many steps, how many meters. And it was really very little. That made me realize that I could probably do a little bit more.*

[17-year-old boy]

*At the moment when it does not provide any benefit to yourself, so if it does not provide that stimulation, I am not sure how fun or motivating it is. … Then, yeah, it does not really offer you anything directly.*

[Father of a 13-year-old girl]

Without clear expectations or a sense of obligation, some children may deprioritize the use of the smartwatch, and the initial excitement may fade. While some children found it challenging to use the smartwatch during intensive treatment phases, others continued to wear it. Uncertainty about its usefulness emerged when children were physically inactive.

*Yeah, only when I received Dexa (dexamethasone) — I found it a bit irritating then, so I did not wear it for a few days. Especially during those 2 or 3 days when I was really at my peak, I took it off for a bit. But after that, I pretty much always wore it again. Yeah, but that was really just because of Dexa — and that kind of speaks for itself, you know. That was the only reason.*

[16-year-old girl]

*Well, it is more like, I wear it mainly because it is practical, and if you are just lying in bed all day, yeah, then it is basically useless.*

[12-year-old boy]

Accurate tracking of physical activity was crucial for maintaining engagement. Children sometimes expressed disappointment when the smartwatch failed to capture their physical activity correctly. In addition, some parents worried that their child might become overly focused on the smartwatch, potentially leading to excessive physical activity or an unhealthy preoccupation with tracking.

*The only downside is that when you are on the treadmill, I think it does not count all the steps — it seems to miss some of them.*

[17-year-old boy]

### Insight and Evaluation

Self-monitoring through the smartwatch provided the opportunity to evaluate physical activity levels and confirmed that the child had been sufficiently physically active. Parents particularly appreciated having insight into their child’s physical activity, with some who used it as an extra tool to assess their child’s health status.

*But it is also helpful for me personally, especially now, because I can start feeling unwell quite quickly — so it is good to be able to see how things are going.*

[16-year-old boy]

*Actually, it was really practical because we saw that suddenly her resting heart rate was much higher and this helped also with, well let’s say, she needed a blood transfusion and probably this was related to her too low hemoglobin level. So it did help us as we thought: ‘Well, her heart rate is really very high’. Yes, so that is really practical. In that way, we can also make use of it to know in time what is going on. … So, it is also, we now have an extra tool. Convenient, right?*

[Mother of a 9-year-old girl]

For children, tracking and sharing physical activity data may be empowering and provide proof that they can remain active despite their illness. Positive reinforcement from their pediatric physiotherapist, enabled by access to their physical activity data, would be valued.

*To prove to myself and others that, even though you are sick, you can still do a lot and you can definitely stay fit.*

[12-year-old boy]

However, the experience of activity tracking was not uniformly positive. While some children found it encouraging, others perceived it as confronting, particularly if their activity levels were low. This perception may discourage using the smartwatch. Children expressed discomfort at the thought of their pediatric physiotherapist having insight into their low physical activity levels and pointing this out.

*I think that if you get a smartwatch at a time like that, and you just cannot reach your goal of the minimum number of steps because you feel too unwell, I think it works counterproductively.*

[Mother of a 9-year-old girl]

### User Experience and Functionality

The design and comfort of the smartwatch influenced its usability during treatment. Most children found the smartwatch attractive and comfortable to wear throughout the entire treatment, while others found the design unattractive or experienced irritation or pressure on their skin. These issues were more likely during early or intensive treatment phases. Factors, such as loose or oversized straps as well as material preferences, impacted wearability.

*Well, it is just — I mean, it is not like it really bothers me. I wear bracelets too, so why would I not be able to wear a watch?*

[16-year-old girl]

*Uh, when I move, it keeps sliding forward and backward, and that was just really annoying.*

[11-year-old boy]

The smartwatch was considered easy to use, though children’s preferences varied. Some children favored a nondigital or visually appealing watch, which may present a barrier to using a smartwatch designed to track physical activity.

Technical limitations also affected the smartwatch’s functionality and reliability. Issues, such as inconsistent visibility of phone notifications, difficulty connecting to digital devices, incorrect setup, improper material, and inaccuracies in physical activity measurements diminished its overall functionality.

### Key Considerations for Long-Term Physical Activity Tracking During Childhood Cancer Treatment

Based on the barriers and facilitators suggested by children and their parents and interpreted by the researchers, the following key considerations are proposed for the future (Table 4):

(1) Personalized approach: A consideration should be made whether the smartwatch is a stimulus or a burden. Daily life feasibility should be discussed, tailoring usage to the individual child and its treatment phases. Providing information on its benefits and relevance may help to reduce perceived burden.

(2) Enhanced persistence: Children who do not perceive a motivational stimulus from the smartwatch should be timely identified, and strategies to wear should be adapted to their needs. Having the ability to set an adjustable, achievable step goal could be motivating for those who fail to reach the lowest daily step goal. Sending motivational and empowering prompts when not being sufficiently physically active might help to reduce negative feelings about sharing physical activity data.

(3) Aligned design and comfort: The smartwatch should align with preferences of children, including color, size, display, strap material, and comfort.

(4) Expanded functionalities: Children suggest additional functions, such as tracking various sports and calling parents, while parents value functions like monitoring temperature.

**Table 4.** Future key considerations for long-term physical activity tracking during childhood cancer treatment gathered during phase 2.

Key considerations and barriers for wearing	Specific considerations
Personalized approach	
Too burdensome for children with low resilience or low physical activity	Consider whether the smartwatch acts as a stimulus or a burden, discuss its feasibility in daily life and tailor its use to each individual child and their treatment phases
High perceived burden by child or parent	Provide information regarding the benefits and relevance of the smartwatch
Enhanced persistence	
No stimulus for physical activity	Identify and guide children who do not perceive motivational stimulus and adapt strategies to needs
Negative feelings about insight into low physical activity	Allow adjustable, achievable daily goal
Negative feelings about sharing insight	Send motivational and empowering prompts by enabling access to children’s physical activity data
Aligned design and comfort	
Unattractive smartwatch	Align the smartwatch with preferences of children, such as features, color, size, and display
Uncomfortable smartwatch	Align the smartwatch with preferences of children, such as size, strap material, and comfort, and consider the effect of treatment on wearing comfort
Expanded functionalities	
Limited features and functionalities for children	Add functions, such as selecting more types of sports or calling
Limited features and functionalities for parents	Add functions, such as measuring additional health outcomes, for example, temperature

## Discussion

### Principal Findings and Comparison With Previous Work

In this study, we showed that long-term physical activity tracking using a smartwatch during childhood cancer treatment was not feasible in our cohort according to the feasibility target we set. Furthermore, we defined barriers, facilitators, and key considerations of real-time and long-term physical activity tracking using a smartwatch in children during cancer treatment.

The result from our study that physical activity tracking using a smartwatch in children with cancer was not feasible is not in line with a previous study in children with acute lymphoblastic leukemia who used a fitness tracker for around 3 weeks during their treatment [27]. This previous study showed that these children were able to consistently use the fitness tracker with low effort. However, 40% (11/28) of eligible patients refused participation, which might induce a potential selection bias. Moreover, the fitness tracker used in the previous study was clipped to clothing rather than a watch worn on the wrist, and the study period was substantially shorter as compared to our study. The authors of the previous study suggested that the use of the smartwatch might only be feasible in a subset of children. Contrary to the results of our study, another previous study in children with a brain tumor reported no adverse effects of using a smartwatch combined with coaching by a physiotherapist for 12 weeks during treatment [26]. However, in the previous study, only children with a brain tumor were included, while in our study, children with a variety of cancer diagnoses were included, which entails a more heterogeneous population. Moreover, the children in the previous study received coaching by a physiotherapist, which was not the case for the children included in our study. Also, inconsistent with the results of our study, during the maintenance phase of childhood acute lymphoblastic leukemia and after treatment for childhood cancer, the daily use of a smartwatch for 6 months was reported to be feasible [28]. Nonetheless, in the previous study, children at the end of their treatment were included while we included a more diverse group of children during different phases of treatment. According to our results, we emphasize the need to consider whether the smartwatch is a stimulus or a burden, discuss daily life feasibility, and tailor usage to the needs and preferences of a child. Furthermore, in our study, tracking physical activity was feasible in some of the children. For the future, it would be of added value to study the characteristics of those who adhered to wearing the smartwatch to find out for which children wearing the smartwatch is feasible.

Besides feasibility and in contrast to the aforementioned studies, our study aimed to identify barriers, facilitators, and key considerations of real-time and long-term physical activity tracking using a consumer-level smartwatch in children during cancer treatment. We found that some children perceived wearing the smartwatch as confronting and felt a pressure to wear it, which may become overwhelming. Although knowledge about wearing experiences of using smartwatches in children with cancer during different phases of treatment is limited, a previous study, exploring the influence of smartwatches on motivation for physical activity in healthy children, reported a potential negative internal pressure to reach a certain step goal, which might have a negative effect on self-image [44]. The authors emphasized the importance of personalizing goals, which is in accordance with our key considerations to apply a personalized approach and tailor the smartwatch to each child individually. Furthermore, empowering children using motivational prompts might help to reduce negative feelings about having and sharing insight. Finally, providing information on its benefits and relevance may aid in reducing perceived burden.

We found that children might perceive the smartwatch in diverse ways, where motivation, value, and expectations play key roles in the success of wearing the smartwatch. These factors not only influence how the smartwatch is perceived but also affect how engaged children and parents feel with the smartwatch. During phase 1, we highlighted the importance of enhancing engagement. Both reducing perceived burden and enhancing engagement are essential for ensuring the smartwatch’s long-term use. Previous literature also mentioned the importance of feeling engaged as a key determinant of the success of physical activity interventions in adolescent and young adult survivors of childhood cancer [36], a smartwatch intervention in children with a chronic health condition [45], or the use of wearable devices to measure physical activity in healthy children [46,47]. For the future, we suggest increasing interest and creating value and meaningfulness by, for example, explaining the possibility of early recognition of aberrant physical activity levels and providing tailored advice and guidance when wearing the smartwatch.

In our study, children reported that the initial excitement of the smartwatch may fade over time. A decrease in the use of a smartwatch during physical activity interventions has earlier been reported in adolescent, young adult childhood cancer survivors [28] and healthy children [29]. Such a novelty effect might be the result of a reduced perceived usefulness. This might consequently have a negative effect on the smartwatch’s wearability in the long term. Based on children’s experiences and previous literature [48,49], we suggest identifying children who do not perceive a motivational stimulus from the smartwatch in a timely manner and adapting strategies to wear it according to their needs. Persistence might be enhanced by, for instance, allowing adjustable, achievable goals for those who fail to reach the lowest daily step goal, sending motivational and empowering prompts when not being sufficiently physically active, and keeping on aligning the smartwatch with the preferences and needs of the child. Moreover, it might be helpful to consider the first period of use as an opportune moment to incorporate the smartwatch into children’s daily living. During this period, children should be informed about the use of the smartwatch, and ideally, this should be continued to encourage them to keep on using it [48-50]. Furthermore, developing and implementing an app that fits with a child’s preferences might be helpful to increase wearability of the smartwatch, especially for those who experienced wearing the smartwatch as overwhelming or confronting.

Children had mixed experiences with wearing the smartwatch during treatment, where attractiveness, comfort, and usability played key roles in its acceptance. These findings are in line with the literature on healthy children [29,50]. We advocate aligning the smartwatch with preferences of children, including color, size, display, strap material, and comfort. Moreover, we suggest incorporating additional functions, as this might increase usefulness. For the future, the importance of personal preferences, comfort, and practical and technical performance should not be underestimated, and appropriate functionality, reliability, and sustainability should be ensured to promote sustained use.

Although a strength of this study is that the feasibility, barriers, facilitators, and considerations of real-time and long-term physical activity tracking using a consumer-level smartwatch in children during cancer treatment were examined using both quantitative and qualitative methods, it does have limitations. First, biases should not be overlooked. Selection bias should be considered, as especially children who are interested in a smartwatch to track their physical activity might have been enrolled. However, in particular, the recruitment rate of 83% for phase 2 undermines selection bias. Confirmation bias should be recognized, as only 1 researcher conducted all interviews. Nonetheless, by using an interview guide and refining the methods and incorporating emerging topics based on children’s experiences, we tried to prevent confirmation bias. Second, the differences in sex and time since diagnosis between children included in phase 1 and 2 might have influenced acceptance as well as the proposed key considerations. Finally, as a qualitative approach has been used, issues of credibility, transferability, and dependability should be acknowledged. We tried to consider these issues by prolonged engagement and persistent observation, thick description, and methodological transparency, respectively [51].

The barriers and facilitators we identified in this study might be helpful for improving the feasibility of physical activity tracking using a smartwatch in children during cancer treatment by acknowledging the key considerations we propose. In addition to these, identifying which children can feasibly wear a smartwatch would provide additional value for them. Furthermore, for future implementation, it would be helpful to explore the fine line between creating a certain commitment without overwhelming children. In addition, understanding how pediatric physiotherapists’ access to children’s physical activity data might be supportive during physiotherapeutic treatment would be valuable. Finally, developing a smartwatch in line with these and other needs of children with cancer and expanding functionalities according to their preferences would be of added value.

### Conclusion

Real-time and long-term physical activity tracking using a smartwatch in children during cancer treatment was not feasible in our cohort. The success of wearing the smartwatch depends on the child’s physical state and the perceived burden by both children and parents. Motivation, perceived value, and expectations play key roles in enhancing persistence, while the balance between positive reinforcement and potential stress from data interpretation affects long-term use. User experiences, including attractiveness, comfort, and usability impact acceptance, emphasizing the essence of a personalized approach, incorporating individual preferences and physical condition, to support adherence.

## Supplementary material

10.2196/75322 Multimedia Appendix 1 Supplementary material feasibility, barriers, and facilitators of long-term physical activity tracking during childhood cancer treatment.

## Abbreviations

HR: heart rate
MREC: Medical Research Ethics Committee
