# Supplementary material for: Feasibility, Barriers, and Facilitators of Long-Term Physical Activity Tracking During Treatment: Interview Study Among Childhood Cancer Patients
Source: JMIR Pediatr Parent. 2025 Aug 27;8:e75322. doi: 10.2196/75322 (PMC12408935; doi:10.2196/75322)
Supplement: Multimedia Appendix 1 [file pediatrics-v8-e75322-s001.docx]

*Section A. Interview guide used during the in-depth interviews in study phase 1.*

**Barriers and facilitators for wearing the smartwatch**

1. Why did you continue wearing the smartwatch? What do you like/enjoy/find useful about the smartwatch?
2. If you had to wear a smartwatch throughout your entire treatment, what should it be able to do or what features should it have for you?
3. What would you think of a smartwatch without a (digital) screen? How would it be if you could only see your activity in the application? What would be the advantages or disadvantages of checking the application instead of looking at a screen on the smartwatch?
4. What were reasons for taking off the smartwatch or not wearing it?

If the smartwatch was taken off or not worn:
a. What would be a solution for you to always wear the smartwatch?

b. How can I help you to wear the smartwatch consistently?

1. Did you wear a watch yourself before this study?

*Section B. Interview guide used during the semi-structured interviews in study phase 2.*

**Reasons for wearing the smartwatch**
Why did you continue wearing the smartwatch? What do you like/enjoy/find useful about the smartwatch?

**Reasons for not wearing the smartwatch**
What were reasons for taking off the smartwatch during the day or not wearing it?

Did you ever forget to wear the smartwatch?

If children forgot to wear the smartwatch:
What would help you remember to wear the smartwatch? How can we help you remember to wear the smartwatch?

**Solutions**
What is helpful for you to keep wearing the smartwatch and not take it off? What solutions are helpful for you?

**Problems**
Did you run into any issues while wearing the smartwatch? What problems did you experience when wearing it?

Do note that the semi-structured interviews conducted during phase 2 were guided by topics raised by children and/or their parents and relevant issues were elaborated on during the interview.

In addition, questions about relevant issues raised during phase 2 (including wearing during the entire treatment, the added value of personalized plan, potentially confronting data and insight by pediatric physiotherapists) were added in all children during the study period.
